# Post introduction evaluation of the malaria vaccine implementation programme in Ghana, 2021

**DOI:** 10.1186/s12889-023-15481-6

**Published:** 2023-03-29

**Authors:** Michael Rockson Adjei, Kwame Amponsa-Achiano, Rafiq Okine, Peter Ofori Tweneboah, Emmanuel Tettey Sally, John Frederick Dadzie, Fred Osei-Sarpong, Michael Jeroen Adjabeng, John Tanko Bawa, George Bonsu, Kwadwo Odei Antwi-Agyei, Basil Benduri Kaburi, Felicia Owusu-Antwi, Elizabeth Juma, Francis Chisaka Kasolo, Franklin Asiedu-Bekoe, Patrick Kuma-Aboagye

**Affiliations:** 1 Ashanti Regional Health Directorate, Ghana Health Service, Kumasi, Ghana; 2 Headquarters, Ghana Health Service, Accra, Ghana; 3 Country Office, WHO, Accra, Ghana; 4 Country Office, PATH, Accra, Ghana; 5 Ghana Field Epidemiology and Laboratory Training Programme, School of Public Health, University of Ghana, Accra, Ghana

**Keywords:** Malaria, RTS, S vaccine, Pilot, Post introduction evaluation, Malaria vaccine implementation Programme, Mosquirix

## Abstract

**Background:**

Malaria remains a public health challenge in Sub-Saharan Africa with the region contributing to more than 90% of global cases in 2020. In Ghana, the malaria vaccine was piloted to assess the feasibility, safety, and its impact in the context of routine use alongside the existing recommended malaria control measures. To obtain context-specific evidence that could inform future strategies of introducing new vaccines, a standardized post-introduction evaluation (PIE) of the successes and challenges of the malaria vaccine implementation programme (MVIP) was conducted.

**Methods:**

From September to December 2021, the WHO Post-Introduction Evaluation (PIE) tool was used to conduct a mixed methods evaluation of the MVIP in Ghana. To ensure representativeness, study sites and participants from the national level, 18 vaccinating districts, and 54 facilities from six of the seven pilot regions were purposively selected. Quantitative and qualitative data were collected using data collection tools that were adapted based on the WHO PIE protocol. We performed summary descriptive statistics on quantitative data, thematic analysis on qualitative data, and triangulation of the results from both sets of analyses.

**Results:**

About 90.7% (49/54) of health workers stated that the vaccine introduction process was smooth and contributed to an overall improvement of routine immunisation services. About 87.5% (47/54) of healthcare workers, and 95.8% (90/94) of caregivers accepted RTS,S malaria vaccine. Less than half [46.3%; (25/54)] of the healthcare workers participated in the pre-vaccine introduction training but almost all [94.4%; (51/54)] were able to constitute and administer the vaccine appropriately. About 92.5% (87/94) of caregivers were aware of the RTS,S introduction but only 44.0% (44/94) knew the number of doses needed for maximum protection. Health workers believed that the MVIP has had a positive impact on under five malaria morbidity.

**Conclusions:**

The malaria vaccine has been piloted successfully in Ghana. Intensive advocacy; community engagement, and social mobilization; and regular onsite supportive supervision are critical enablers for successful introduction of new vaccines. Stakeholders are convinced of the feasibility of a nationwide scale up using a phased subnational approach taking into consideration malaria epidemiology and global availability of vaccines.

## Background

Malaria is a life-threatening disease caused by five species of mosquito-transmitted parasites in humans [1]. In 2020, an estimated 241 million cases of malaria were recorded globally with 627,000 deaths. Children under five years are the most vulnerable and accounted for 80% of the mortalities, of which Africa accounted for 95% of cases and 96% of deaths [2].

In Ghana, malaria accounts for approximately 30% of outpatient attendance and 23% inpatient admissions [3]. *Plasmodium falciparum* is the predominant malaria parasite in Ghana, accounting for approximately 80–90% of severe morbidity and mortality [4]. Generally, there is a stable transmission pattern. Studies conducted in 2014 and 2019 have shown endemicity ranging from intermittent transmission in the Greater Accra Region to intense seasonal transmission in the Upper West Region and seasonal transmission in the rest of the country [4, 5]. Ghana has made significant strides in malaria prevention and control with existing interventions, contributing substantially to a reduction in malaria-related mortalities [5]. However, the disease burden remains significant [4], and deployment of additional tools may facilitate achievement of elimination status.

A well tolerated and effective vaccine with an acceptable safety profile could be a potentially important intervention for malaria control. In January 2016, Ghana (as well as Kenya and Malawi) responded to the World Health Organization (WHO) call for national Ministries of Health to express interest in collaborating in the RTS,S/AS01 malaria vaccine pilot implementation programme, and was reaffirmed in March 2016. Approval of the country’s request was announced by WHO (alongside with Malawi and Kenya) in April 2017 [6]. The pilots were designed to assess the feasibility of delivery, safety and impact on disease morbidity and mortality in the context of routine use alongside other currently recommended malaria control measures [7].

The malaria vaccine was introduced into the national childhood immunisation programme from May 2019 [8]. Based on the positive results from the pilots in Ghana, Kenya, and Malawi, in October 2021, the WHO advisory bodies for immunisation and malaria recommended the use of the RTS,S/AS01 malaria vaccine for the prevention of P. falciparum malaria in children living in areas of moderate to high transmission as defined by the WHO. There is also strong evidence of high efficacy of the vaccine using seasonally timed vaccinations in areas where malaria transmission is highly seasonal [7].

Although the Ghana Health Service (GHS) and partners conducted routine monitoring and supportive supervision as part of the introduction process, a standardized post-introduction evaluation (PIE) expected to be carried out six to 12 months after the vaccine introduction [8] was delayed due to competing activities in the wake of the COVID-19 pandemic. The main objective of this evaluation was to assess success and challenges of the malaria vaccine implementation programme (MVIP) and to provide recommendations for introduction of new vaccines in the future.

## Methods

### Malaria vaccine implementing areas

Ghana had 10 regions until 2019 when six new ones were carved out of four of the old regions [9]. Presently, the country has16 regions and 261 districts. The total population according to the 2021 population and housing census was 30.8 million with children under five years constituting approximately 20%.

**Fig. 1:**
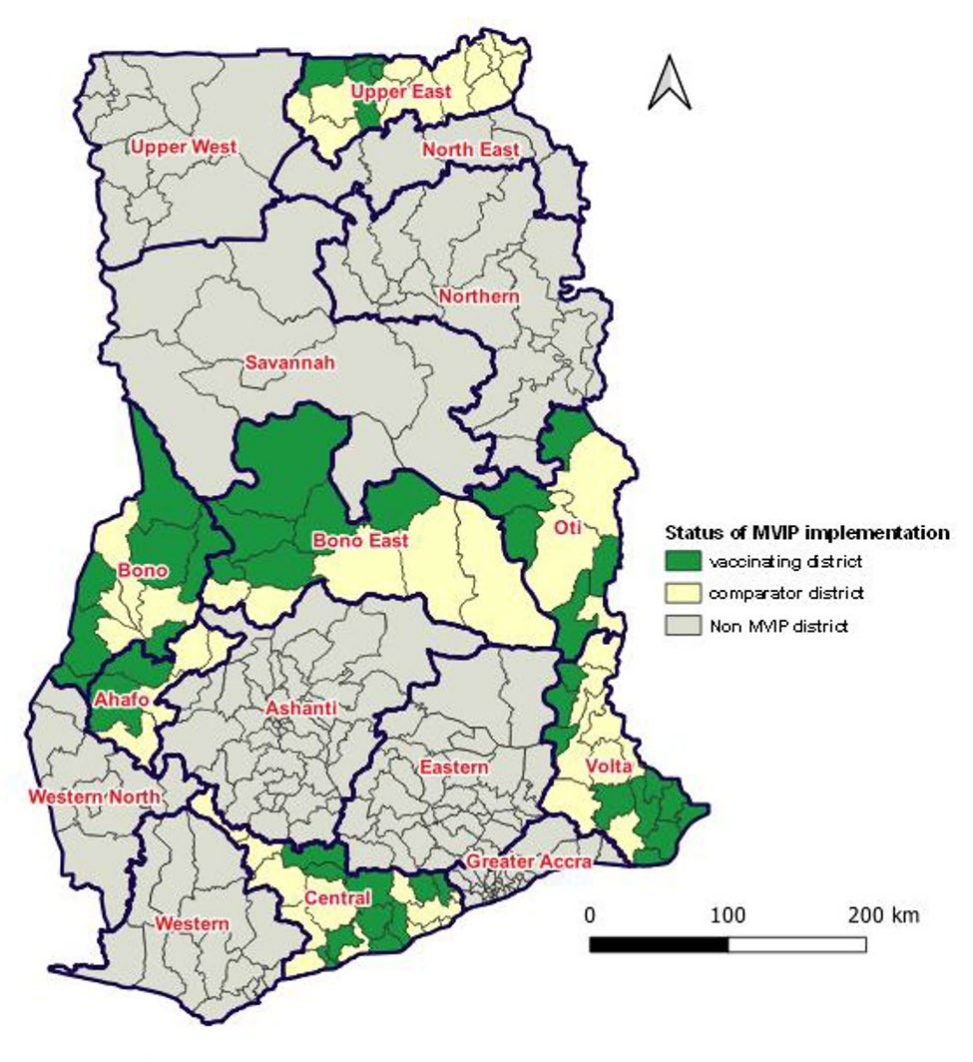
Geographical distribution of pilot districts for malaria vaccine introduction, Ghana. Source: EPI, 2022 (MVIP bulletin, Ghana, 2022; unpublished)

The vaccine was introduced in 42 out of 93 districts in Brong Ahafo (now divided as Bono, Bono East, and Ahafo), Central, Volta (presently split as Volta and Oti), and Upper East regions (Fig. 1). The selection of the regions for the pilot programme was based on the criteria recommended by the Ghana Malaria Vaccine Technical Working Group: These included: high malaria burden (PfPr ≥ 20%); strong EPI performance, malaria ecological zone, rural/urban mix; and required number of age-eligible children or target population to receive the vaccine.

Six of the 42 districts involved in the GSK-led Phase IV observational study were excluded in the post-introduction evaluation. The Upper East Region was not selected because its implementing districts (two in all) were part of the Phase-IV study. The remaining 51 districts in the pilot regions served as comparator sites for the purpose of the programme evaluation.

### Study design

The WHO Post-Introduction Evaluation (PIE) tool [8] was adapted and a mixed methods study was carried out from September to December 2021. The PIE tool is a standard protocol that provides a systematic method for evaluating the outcome of a vaccine introduction on the existing immunisation system in a country.

It is designed for immunisation managers in countries that have introduced new or underutilized vaccines. It focuses on a range of programmatic aspects, such as pre-introduction planning, vaccine storage and wastage, logistics of administering the vaccine, and community receptiveness to the vaccine.

### Sampling of study sites and participants

Study sites were purposively selected using pre-defined criteria (vaccination coverage, geographical settings, and regional representativeness) aligned with the recommendations in the WHO PIE Protocol. The selection criteria included setting (rural, peri-urban, and urban); immunisation coverage (high and low uptake of 2020 RTS,S 1 using a coverage of 70% as cut off); and geographical spread (study sites were equitably selected from all implementing regions).

**Fig. 2:**
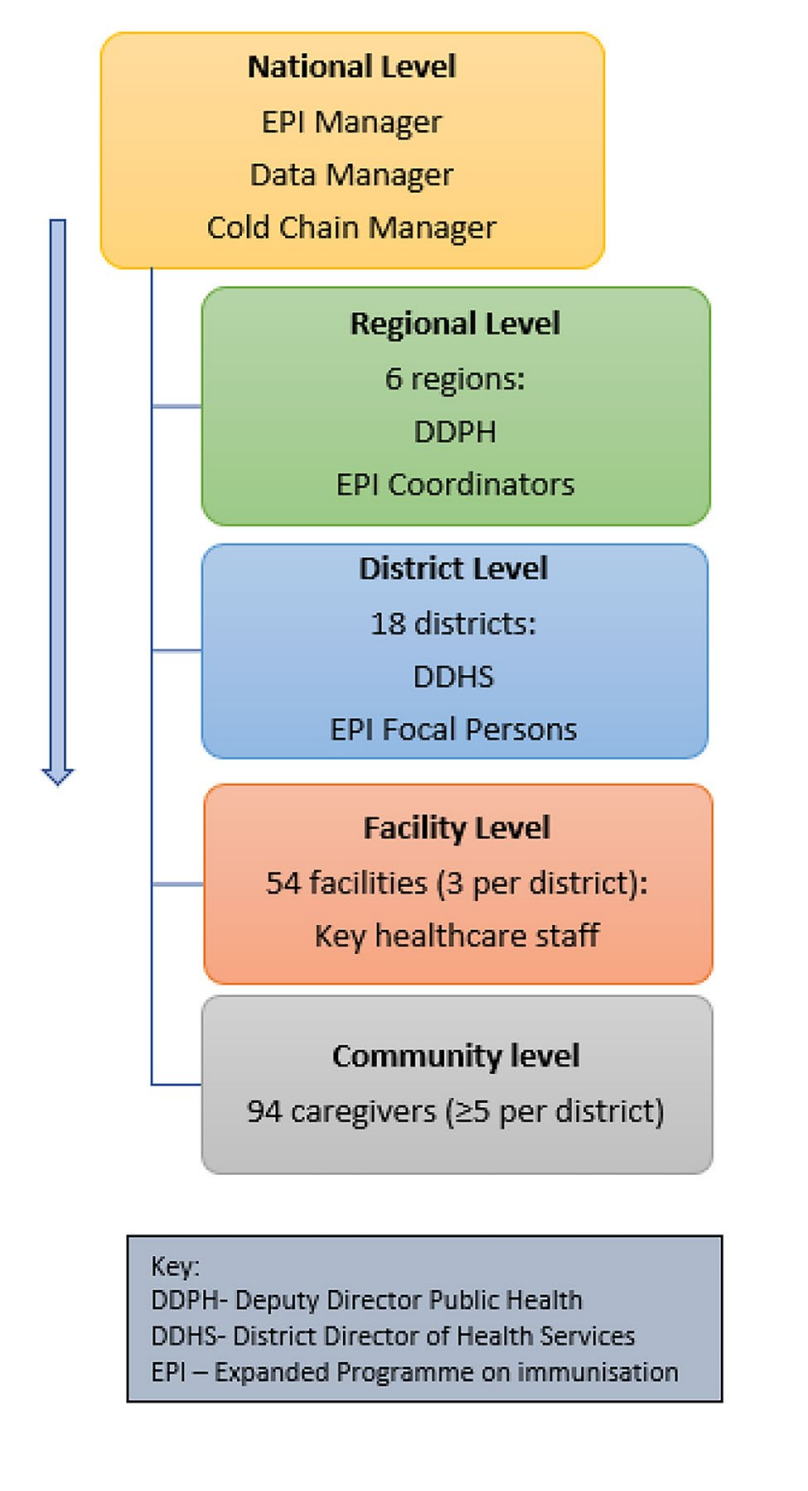
Selection sequence of respondents, malaria vaccine PIE, Ghana

The national level, six regions, 18 districts and 54 facilities, and 94 caregivers were selected for the evaluation (Fig. 2). At each level health staff were purposively selected based on their functions, and caregivers were conveniently sampled. The selection of study sites and participants was based on the recommendations from the PIE protocol.

### Data collection

Data collection was supported by trained research assistants with overall process coordinated by the independent evaluation consultant. More than 10 documents including national immunisation schedule, malaria vaccine operational guideline, malaria vaccine training guide, education and communication materials, malaria disease surveillance bulletin, malaria vaccine introduction plan, malaria vaccine regional review reports, vaccine safety guideline and reporting form, Ghana malaria vaccine technical brief summary, RTS,S Ghana factsheet, and malaria vaccine implementation quarterly bulletin were reviewed by the evaluation team to provide context to the malaria vaccine and its introduction in Ghana.

Questionnaires and interview guides were administered to healthcare workers at the national, regional, district, and facility levels by trained research officers and the PIE consultant. The questionnaires collected information on pre-implementation planning, coverage, recording and reporting, training, supportive supervision, demand generation, safety monitoring, cold chain, and waste management. The interview guides gathered views on impact of vaccine introduction on service delivery and child health. The caregiver questionnaire focused on vaccine awareness, service experience and knowledge of dose schedule. All responses were recorded. We inspected cold and dry vaccine storage areas as well as observed service delivery at vaccination points. Open Data Kit (ODK) was used to collect data for the survey.

### Data analysis

Quantitative data were extracted onto Microsoft Excel 14.0 spreadsheet and exported into Epi Info statistical software (Epi Info version 7.2.2.16, www.cdc.gov>epiinfo) for analysis. Summary descriptive statistics were generated for the quantitative data. Inductive coding and thematic analysis were performed on the qualitative data. Results were then triangulated from both sets of analyses. Where there were conflicts of thematic classification of data, it was resolved through team discussion and consultation with other field experts.

## Results

### Pre-implementation planning, coverage, drop-out, recording and reporting

**Fig. 3:**
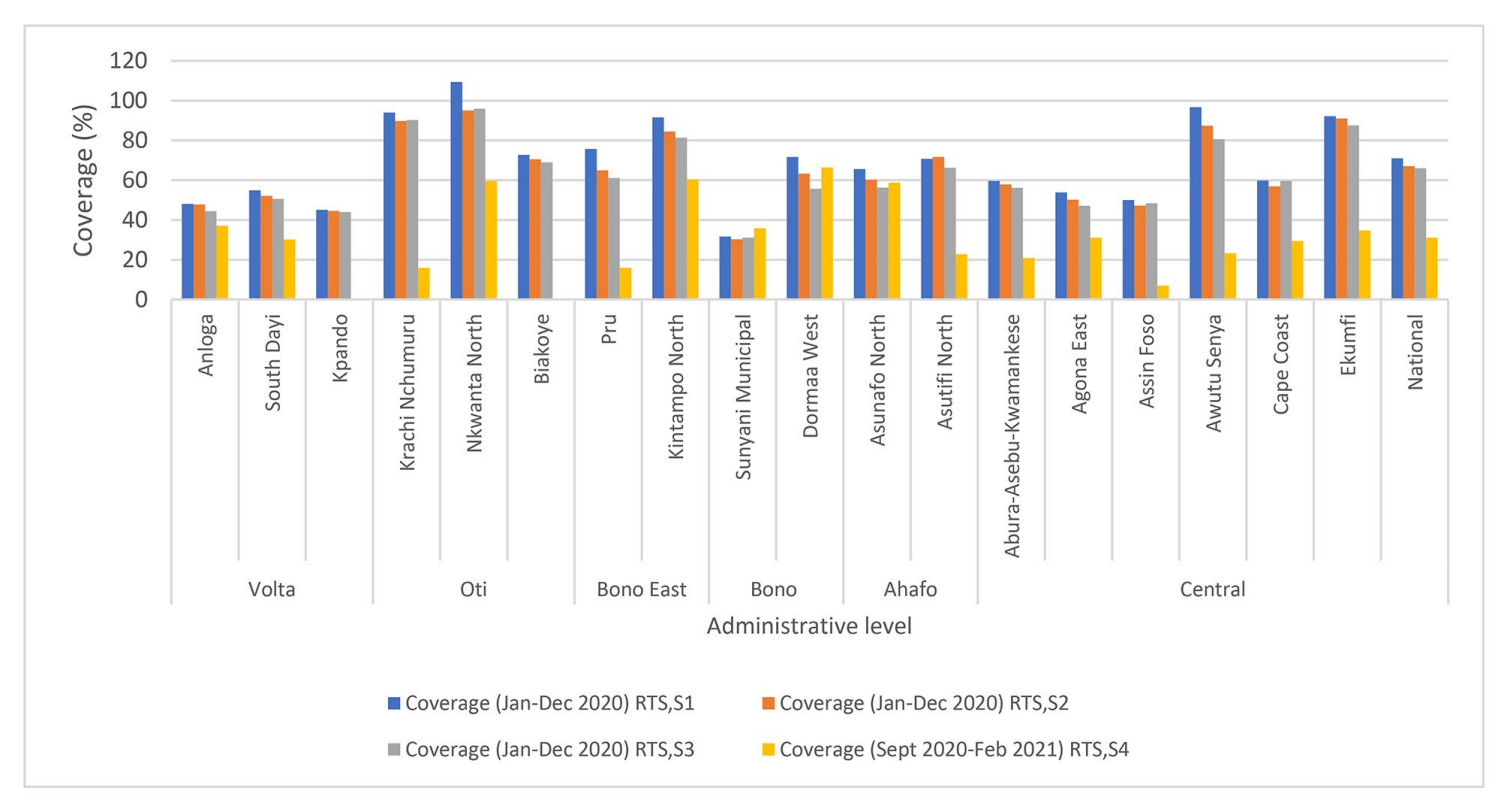
Coverage of RTS,S doses 1, 2, 3, and 4 by district, Ghana; 2020–2021

In all the pilot regions and districts, there were updated immunisation registers and data collection tools that incorporated columns for RTS,S. 83% [83%; (45/54)] of the facilities reported RTS,S vaccination data monthly to the next level and 77.8% (42/54) of the staff interviewed knew the accurate formula for calculating coverage. Generally, the coverage of RTS,S decreased sequentially from the first dose to the fourth in all the districts (Fig. 3). Only 22% (4/18) of districts had RTS,S 1 and RTS,S 3 dropout rate below 10%.

### Training and knowledge of healthcare workers

Less than half [46.3%; (25/54)] of the vaccinating staff received pre-vaccine introduction training but almost all [94.4%; (51/54)] were able to constitute and administer the vaccine appropriately.

### Supportive supervision

Although 87% (47/54) of facilities benefited from technical supportive supervisory visits in the preceding six months, only 14.9% (8/54) had written feedback. Majority [88.8%; (16/18)] of district level staff had accurate knowledge of the formula for wastage calculation and same proportion of districts had wastage rate below 25%.

### Advocacy and vaccine acceptance

**Fig. 4:**
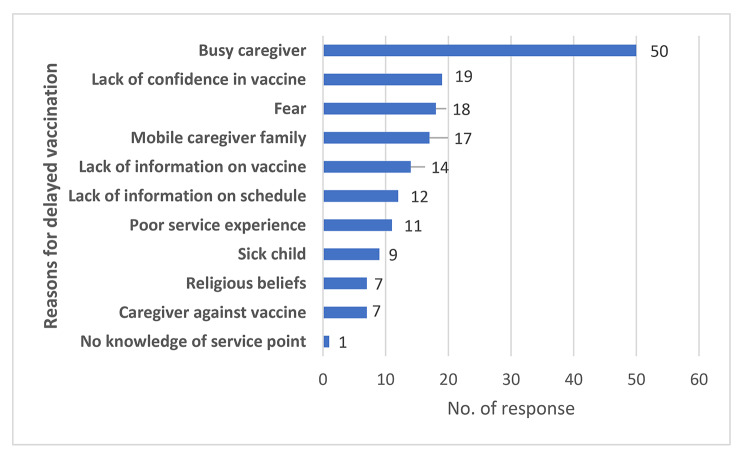
Caregiver factors influencing vaccine uptake

**Fig. 5:**
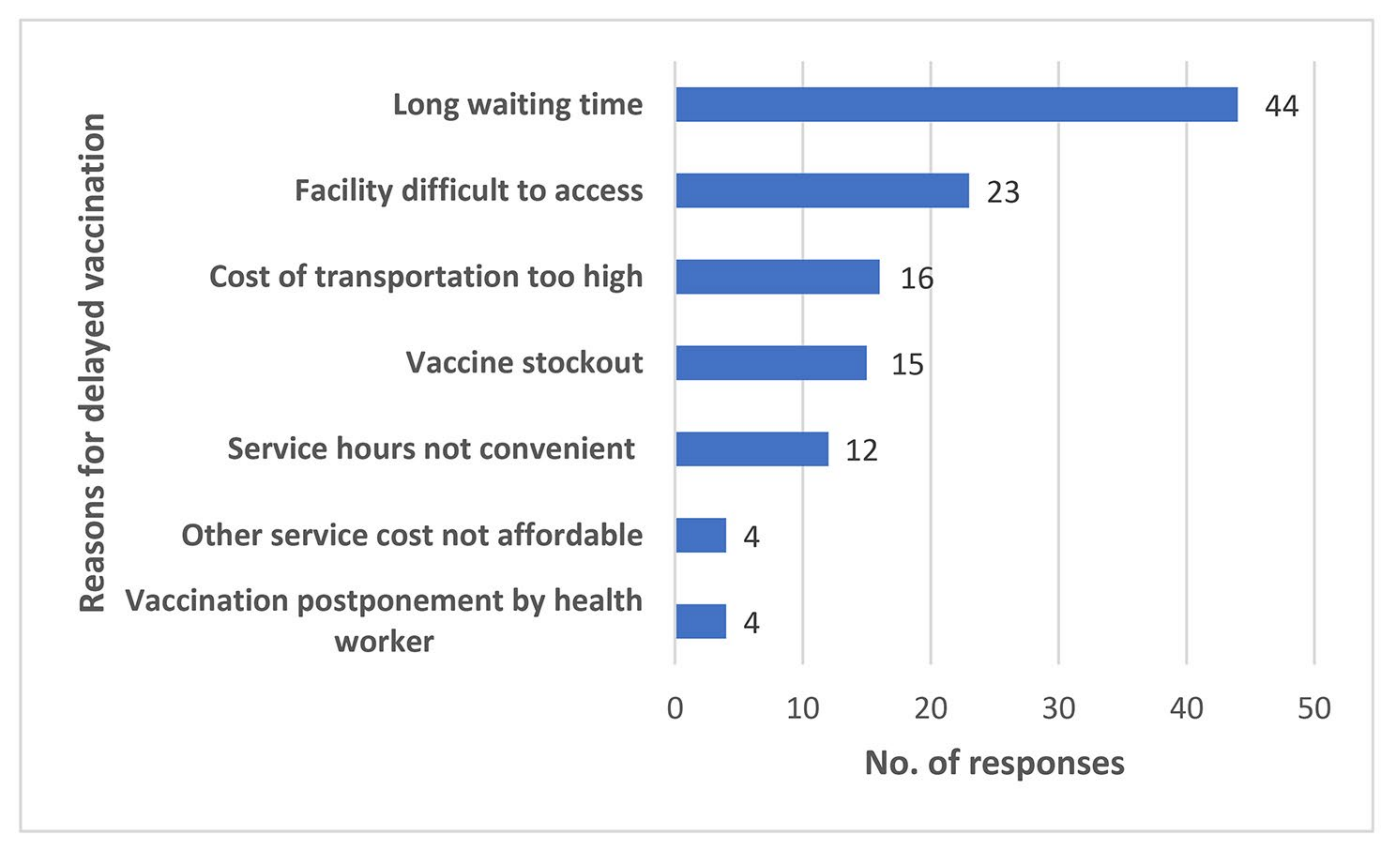
Facility factors influencing vaccine uptake

Majority [92.5%;(87/94)] of caregivers were aware of the RTS,S introduction but only 44% (41/94) had accurate knowledge on the number of doses needed for maximum protection. More than 80% [87.5%; (47/54)] and 95.8% (90/94) of healthcare workers and caregivers respectively accepted RTS,S. The main reasons for delayed vaccination were ‘busy caregiver’ (Fig. 4) and long waiting time at service delivery points (Fig. 5).

### Vaccine safety monitoring

More than half [55.6%; (30/54)] of the facilities had forms for reporting adverse events following immunisation (AEFI) and adverse event of special interest (AESI) but only 22.2% (12/54) had detected at least one AEFI since introduction of RTS,S. About nine out of 10 [91.8%; (86/94)] caregivers were able to mention at least one possible reaction/side effect of the vaccine. There was an observed increase in adverse event reporting following RTS,S introduction.

### Cold chain, injection safety, and waste management

About 39.0% (7/18) of the districts made changes to the cold chain and vaccine storage system prior introduction of RTS,S and 83% (45/54) of facilities had functional refrigerators. Less than one-third [27.8%; (5/18)] of districts made adjustment in the waste disposal system and 77.8% (42/54) of the facilities had access to incinerator. About 24% (13/54) reported RTS,S stockout in the preceding six months and 3.8% (4/54) had unusable vaccines because they were either expired or at vaccine vial monitor (VVM) stage 3 or 4. Of the 18 districts, 16 had adequate capacity for clean storage of vaccines and vaccine administration equipment.

### Effect of vaccine introduction on service delivery

Ninety percent (90%)  of the facilities had ever cancelled critical routine activities due to lack of funds. To this a staff (Field Technician, Male) said: *“Sometimes we are unable to get money to purchase fuel and had to abandon outreach services. This results in default especially among children living in the hard-to-reach communities”*.

Majority [90.7%; (49/54)] of health workers affirmed that the process was smooth, and this was supported by the response of an interviewee (National Level Staff, Male) who said: *“we did not encounter any challenges in the roll out except that uptake in the initial phase was poor due to hesitancy. However, with continual engagement this has improved”*.

Health workers believed that the vaccine introduction has improved immunisation service. A regional level officer (female) had this to say: *“some of our facilities did not have vaccine fridges and motor bikes but thanks to MVIP these logistics have been made available”.* On the effect of the vaccine on health of children, a district level staff (male) said: “*the number of malaria cases recorded monthly in this district has reduced by about one-fifth. Caregivers are seeing the impact and are more encouraged to vaccinate their children”*.

## Discussion

Substantial investment was made in improving cold chain and transport; capacity building; and social mobilization before and during the pilot. The account of health workers and data from some facilities indicate that the vaccine has contributed to improved under-five malaria morbidity and strengthened immunisation service delivery through bridging cold chain logistics and transport gaps in the implementing districts.

Given that malaria is an important cause of morbidity and mortality among children under five, public acceptance of a malaria vaccine was highly anticipated, but this was not observed in the initial phase of the vaccine introduction. The caregiver reluctance observed in the initial phase of introduction was also documented in Malawi (MVIP PIE, Malawi, 2021; unpublished). The existing caregiver confidence in the EPI might have introduce some complacency in the timing and intensity of advocacy, community engagement, and social mobilization activities given that one of the key driving factors of successful vaccine roll out is timely stakeholder involvement [10]. Although approval of the country’s request for RTS,S was received in 2017, active engagement of caregivers started just around the time of implementation in 2019. In this long intervening period, the information gap was filled with rumours conveyed through social media and other information platforms [11].

Apart from misinformation and disinformation, other factors contributed to the slow uptake of the vaccine. Poor health worker understanding of the eligibility criteria and dosing schedule resulted in missed opportunities as some eligible children were denied service. Additionally, some caregivers relocated to non-implementing areas on socioeconomic grounds with consequent truncation of the vaccination schedule. Literacy, knowledge of immunisation, attitude of caregivers, and geographic mobility are among key factors that significantly impact vaccine uptake [12–14]. Some caregivers accepted all vaccines except RTS,S although it was available at the vaccination centres during the peak of the rumours and disinformation. This position was partly instigated by some health workers who specifically asked caregivers if they wanted their children to receive RTS,S despite same not inquired for other vaccines. Caregivers trust judgment of health workers and uncertainties in communication negatively impact uptake of vaccines [15].

According to the EPI schedule of Ghana, children complete all routine vaccines (for those who do not default) by 18 months [16] and many caregivers often do not attend child welfare clinics thereafter, although vitamin A is administered every six months and growth monitoring continues till, they turn five years. This affected uptake of the 4th dose which was scheduled at 24 months. The comparatively low 4th dose uptake also highlights challenges with optimizing uptake of child health interventions in the second year of life and calls for novel strategies including integration of health interventions to strengthen delivery. The dropout rate for RTS,S 1 and RTS,S 3 has been below 10% and this emphasizes the strong performance of the immunisation programme particularly in the first year of life although there are regional and district disparities. Occasional shortage of RTS,S vaccines at the facility level due to local distribution challenges in some districts affected availability and resulted in missed opportunities for vaccination [17].

The existence of system for continuous staff training is commendable given that nine out of 10 of the staff constituted and administered the vaccine appropriately despite the high attrition of staff trained prior to the vaccine introduction. Peer-to-peer knowledge transfer, supportive supervision and on-the-job coaching, and availability of job aids were among key interventions instituted to ensure staff have adequate knowledge on the vaccine and its management.

The relatively improved reporting of adverse events in the pilot districts was because of the strengthened vaccine safety surveillance following capacity building of health workers at all levels as part of the vaccine introduction [18]. Building capacity of health workers and providing them with appropriate incentives (equipment, recording tool, transport, etc.) improves detection and reporting [19, 20] of adverse events although many of the cases are often coincidental [21]. Majority of the reported AEFI were non-serious and the frequently included fever, headache, cough, abdominal pain, and diarrhoea. Apart from fever, the others were not noted frequently, and this is consistent with the findings from the phase III clinical trial of the vaccine [22].

The intensification of public education, rumour management, and advocacy soared up caregiver confidence, and vaccination coverage is consistently improving. This has also been facilitated by targeted supportive supervision and periodic intensification of immunisation activities in the implementing districts. The schedule for the fourth dose has been reviewed from 24 months to 18 months following recommendations by the National Immunisation Technical Advisory Group (NITAG). There is also planned extension of vaccine introduction to the non-vaccinating district (51 in all) in the implementing regions by first quarter of 2023. These new developments will help to address the challenges of uptake and bring coverage up to par with other routine vaccines.

A limitation of the study is that not all implementing districts were included in the evaluation and the findings might not be reliably generalizable for the country. However, this was mitigated using robust selection criteria that ensured representation of all district-groupings with reference to geographical location, coverage, and service delivery enablers and challenges.

## Conclusions

The malaria vaccine has been piloted successfully in Ghana and plans are underway to scale up implementation in other parts of the country. Although the initial phase was characterized by low vaccine uptake due to caregiver hesitancy, coverage has improved steadily following intensive advocacy, community engagement, and social mobilization; and onsite supportive supervision. Adequate cold chain capacity; timely and effective advocacy, community engagements, and social mobilization; alignment with existing routine immunisation schedules (convenience of dose schedules); robust defaulter tracing mechanism; effective healthcare worker and community education; and regular supportive supervision are among key factors worth considering prior to a nationwide scale up. The aforementioned factors generally underpin robust immunisation systems and the lessons learned from the MVIP are applicable in strengthening immunisation programmes for other vaccine-preventable diseases.

## Data Availability

The datasets generated and/or analyzed during the study are not publicly available because it is not possible to fully anonymize the transcript files. The experiences shared by the respondents are intrinsically shaped by their specific roles and responsibilities within the implementation making it possible for linkage of responses to respective identities. However, they will be available from the Ghana Health Service through the corresponding author upon reasonable request.

## List of abbreviations

AEFI: Adverse Event Following Immunisation
AESI: Adverse Event of Special Interest
EPI: Expanded Programme on Immunisation
EMA: European Medicine Agency
GHS: Ghana Health Service
GSK: GlaxoSmithKline
MVIP: Malaria Vaccine Implementation Programme
ODK: Open Data Kit
PIE: Post Introduction Evaluation
WHO: World Health Organization
